# Anomalous photoluminescence in InP_1−x_Bi_x_

**DOI:** 10.1038/srep27867

**Published:** 2016-06-13

**Authors:** Xiaoyan Wu, Xiren Chen, Wenwu Pan, Peng Wang, Liyao Zhang, Yaoyao Li, Hailong Wang, Kai Wang, Jun Shao, Shumin Wang

**Affiliations:** 1 State Key Laboratory of Functional Materials for Informatics, Shanghai Institute of Microsystem and Information Technology, CAS, 865 Changning Road, Shanghai 200050, China; 2 University of Chinese Academy of Sciences, Beijing 100049, China; 3 National Laboratory for Infrared Physics, Shanghai Institute of Technical Physics, Chinese Academy of Sciences, 500 Yutian Road, Shanghai, 200083, China; 4 Shandong Provincial Key Laboratory of Laser Polarization and Information Technology, Department of Physics, Qufu Normal University, Qufu 273165, China; 5 Department of Microtechnology and Nanoscience, Chalmers University of Technology, 41296 Gothenburg, Sweden

## Abstract

Low temperature photoluminescence (PL) from InP_1−x_Bi_x_ thin films with Bi concentrations in the 0–2.49% range reveals anomalous spectral features with strong and very broad (linewidth of 700 nm) PL signals compared to other bismide alloys. Multiple transitions are observed and their energy levels are found much smaller than the band-gap measured from absorption measurements. These transitions are related to deep levels confirmed by deep level transient spectroscopy, which effectively trap free holes and enhance radiative recombination. The broad luminescence feature is beneficial for making super-luminescence diodes, which can theoretically enhance spatial resolution beyond 1 μm in optical coherent tomography (OCT).

Recently, bismuth (Bi) containing semiconductors have attracted increasing interest due to their intriguing properties and potential applications in long wavelength and energy efficient optoelectronic devices. It has been reported that when the Bi concentration increases from doping level to dilute alloy, the interaction of the Bi 6p electron bounding orbital with the valence band (VB) maximum results in both a large bowing effect in the band gap energy^1^,^2^,^3^,^4^,^5^,^6^ and a large spin-orbit (SO) splitting^3^,^5^,^6^. It is well known that the crystal quality of III–V semiconductors is highly affected by the growth temperature. A low growth temperature is undesirable as it typically leads to increase of defect densities and optical quality degradation. Besides, accurate control of Bi flux to avoid forming Bi droplets and use of a low growth temperature to incorporate a significant fraction of Bi are required during epitaxial growth^7^,^8^,^9^,^10^,^11^,^12^. Thus, there are many challenges for growing high-quality bismide alloy.

While most experimental studies are focused on dilute GaAsBi^13^,^14^,^15^,^16^,^17^, GaSbBi^18^,^19^,^20^,^21^,^22^,^23^,^24^,^25^, InSbBi^26^,^27^,^28^,^29^ and InAsBi^30^,^31^,^32^,^33^,^34^,^35^,^36^,^37^, we have recently reported the first successful growth of high quality InPBi single crystal thin films by gas source molecular beam epitaxy (GSMBE) and investigated the effect of rapid thermal annealing on the structural and optical properties of InP_1−x_Bi_x_. They show very strong and broad photoluminescence (PL) at 300 K^38^,^39^ and good thermal stability for device application^40^. As reported previously, the PL signals of most bismide correspond to the band-to-band transitions and the band gap decreasing rate with increasing Bi can be extracted from the PL spectra. Mazzucato *et al*. and Tiedje *et al*. reported the red shift of the GaAsBi PL peak energy of about 75 meV/%Bi^41^ and 85 ± 10 meV/%Bi^42^, respectively. Ma *et al*. reported that the InAsBi PL peak energy decreases with increasing Bi concentration at a rate of 55 meV/%Bi^30^. Kopaczek *et al*. reported the redshift of GaSbBi band gap emission of about 29 meV/%Bi^21^. All these results are in agreement with the theoretical calculations by Polak *et al*.^43^. While the PL of InPBi is anomalous compared to other bismide alloys, the strong and broad PL signals reveal transition energy much smaller than the predicted InPBi band gap as well as the extrapolated band edges from absorption measurements. The PL intensity is about two orders of magnitude higher than that of InP thin films grown under the same condition. The full width at half maximum (FWHM) is nearly 700 nm in the spectral range of 1.4–2.7 μm^38^,^39^. This abnormal feature has potential applications for making super-luminescence diodes which can theoretically enhance spatial resolution beyond 1 μm in optical coherent tomography (OCT).

In this paper, we provide a detailed study of the PL evolution of InP_1−x_Bi_x_ with Bi concentration up to 2.49% and unveil the physical origin of the anomalous PL signals. The broad PL consists of several well resolved peaks which are all related to deep levels in InPBi.

## Results

### Structural properties

**Figure 1:**
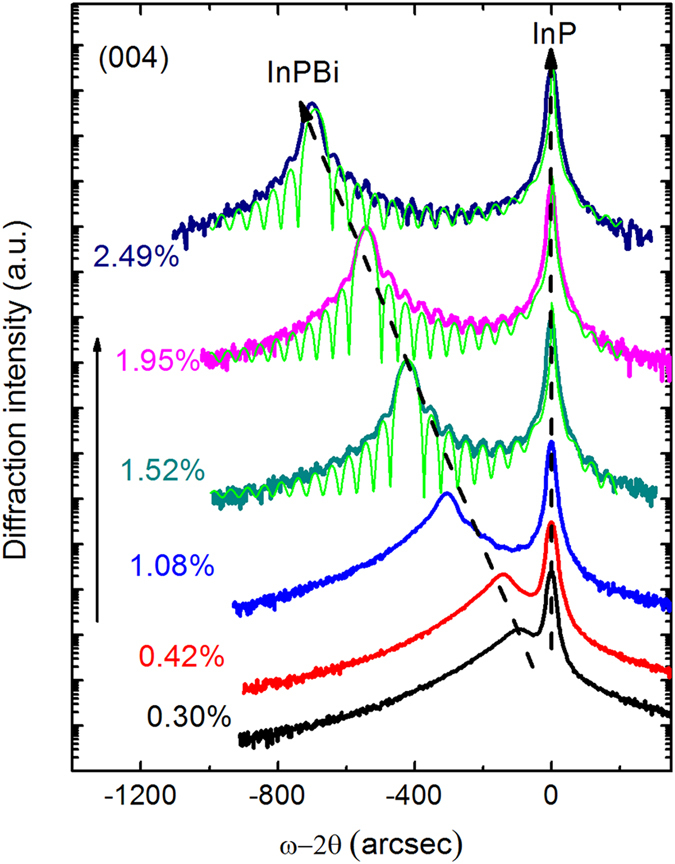
HRXRD (004) ω-2θ rocking curves of InP_1−x_Bi_x_ samples with various Bi concentrations. The three green lines are simulations using the dynamical theory.

Figure 1 shows HRXRD (004) ω-2θ rocking curves of InPBi with various Bi concentrations and the FWHM extracted from Fig. 1 is listed in Table 1. The right narrow peak with a FWHM of around 20 arcsec corresponds to the InP substrate while the left peak corresponds to the InPBi layer. The Bi concentration is determined by two symmetric (004) scans and two asymmetric (115) scans. All the samples are found fully strained within an experimental error of ±0.2%. When only doped with 0.3% Bi, a broad InPBi layer peak with a FWHM of 189 arcsec emerges to the left of the InP substrate peak. The broad epilayer peak indicates the existence of significant alloy fluctuations introduced by Bi doping. As Bi concentration increases, the InPBi peak moves toward low angles and becomes narrow with a FWHM decreasing to 46 arcsec, revealing good crystal quality for such a thin layer. Meanwhile, the Pendellösung fringes suggest good InPBi/InP interface for the samples with x = 1.52–2.49%. Simulations of the rocking curves reveal an average film thickness of 384 nm, in agreement with the nominal thickness value of 390 nm. It is well known that Bi has a surfactant effect when growing III–V alloys. The existence of Pendellösung fringes for x ≥ 1.52% indicates that the surfactant effect is effective only when the growing surface is covered with enough Bi atoms.

### Optical properties

**Figure 2:**
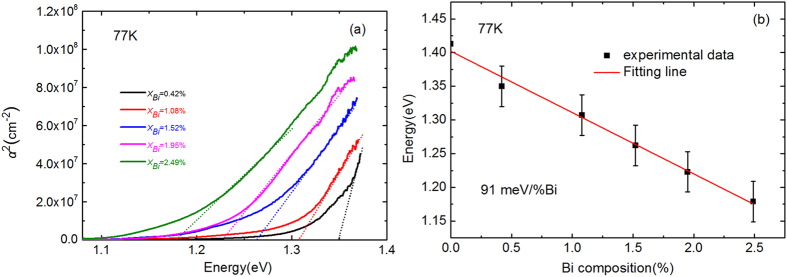
(**a**) Square of absorption coefficient of InPBi samples with various Bi compositions as a function of photon energy at 77 K. (**b**) Band gap energy of InPBi measured from absorption spectra as a function of Bi composition. The error bars of the experimental data are labeled. The solid line is the linear fitting line of the experimental data.

Figure 2(a) shows square of absorption coefficient of InPBi films with various Bi compositions as a function of photon energy at 77 K. The band gap value is obtained from the linear extrapolation of the rising part for each sample. As Bi composition increases, the band edge of InPBi shifts to longer wavelengths revealing a reduction of the band gap energy. As shown in Fig. 2(b) the Bi-induced band gap reduction is about 91 meV/%Bi.

**Figure 3:**
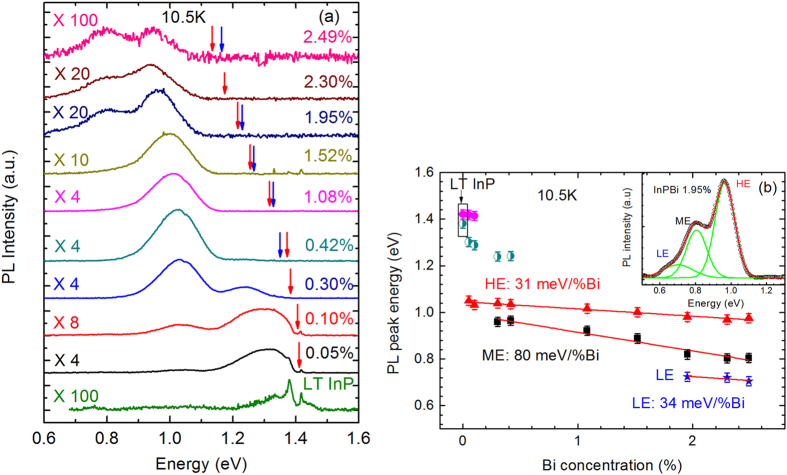
(**a**) PL spectra of InP_1−x_Bi_x_ samples with various Bi concentrations and LT InP reference sample at 10.5 K. Red arrows point the theoretical band gap values of InPBi following the 106 meV/% Bi^44^. Blue arrows point the band gap measured from absorption spectra. (**b**) PL peak energy evolution versus Bi concentration. The left upper dots are related to the recombination of excitons bounded to shallow donors and the Bi-related no-phonon transition. The red solid line is a linear fit of HE (slope = 31 meV/%Bi); the black solid line is a linear fit of ME (slope = 80 meV/%Bi); the blue solid line is a linear fit of LE (slope = 34 meV/%Bi). The inset shows a typical spectrum of InP_1−x_Bi_x_ (*x* = 1.95%). The dotted curve is the measured spectrum. The green dashed curves are the Gaussian peak fitting. The red solid curve is the sum of the fitted lines.

Figure 3(a) shows PL spectra of the InP_1−x_Bi_x_ samples with various Bi concentrations (0.05% ≤ *x* ≤ 2.49%) and the LT InP reference sample measured at 10.5 K. The spectra are magnified by particular factors for visually suitable in height. In general, the PL emission features shift to lower energy with increasing Bi concentration as expected, and the spectral line-shape also changes. Red arrows point the theoretical band gap values of InPBi following the 106 meV/% Bi^44^. Blue arrows point the band gap measured from absorption spectra shown in Fig. 2. The LT InP reference spectrum manifests two sharp peaks at about 1.42 eV and 1.39 eV, respectively, superimposed by a broad peak centered at about 1.3–1.35 eV. The InPBi samples have totally different PL characteristic compare to that of the InP grown at the same condition. In all cases, the observed PL signals show peak energies much smaller than the band gap of InPBi shown by the arrows in Fig. 3(a). When only doping with 0.05% Bi, a strong and broad feature appears at about 1.30 eV in addition to the two sharp peaks also found in the reference LT InP sample, and becomes a dominant signature in the PL spectra. Also a weak and broad peak at about 1.05 eV appears. The 1.30 eV peak gradually shifts to lower energy and diminishes in intensity, and eventually disappears for x = 0.42%, while the other 1.05 eV peak gradually dominates the PL spectrum with increasing Bi up to 1.08%. As Bi content increases to 1.52%, the PL intensity begins to decrease and a new feature at around 0.8 eV emerges. This trend persists when further increasing Bi concentration and the 0.8 eV peak further evolves to two peaks that will be discussed below. Overall, the InPBi PL spectral evolution versus Bi concentration shows that features at high energy gradually quench and features at low energy emerge. Such anomalous PL behaviors as a function of Bi concentration are different from those found in GaAsBi^42^ and GaSbBi^21^ where PL peak follows the band gap reduction for small amount of incorporated Bi composition.

Two spectral regions are defined: above and below 1.15 eV. The PL features above 1.15 eV are related to the case of very dilute Bi doping in InP. Similar results were reported in Bi-doped GaP^45^,^46^ and InP^47^ where sharp PL peaks close to the band gap of the host materials together with a broad band lying within the 250 meV below the band gap are clearly observed at low temperatures. The broad band consists of many well resolved peaks which are assigned to phonon replica of the Bi related peak. The 1.42 eV peak was attributed to the D^0^ line due to the recombination of excitons weakly bounded to shallow donors, while the 1.39 eV peak was assigned to the Bi-related no-phonon transition and labeled as Bi^0 ^47. In our case, fine structures related to phonon assisted recombination are not resolved, probably due to the low spectral resolution and weak signals. The most interesting and anomalous PL phenomena are the transitions with energy far below the band gap of InP, i.e. below 1.15 eV. In the rest of this paper, we will focus on results and discussions on these PL signals.

The broad and asymmetry PL features indicate existence of multi-peaks. So Gaussian fitting was implemented to resolve these multi-peaks and acquire peak energy, intensity and FWHM of each peak quantitatively. The inset of Fig. 3(b), for example, shows a typical fitted PL spectrum of InP_1−x_Bi_x_ with *x* = 1.95%. Three peaks are well resolved and labeled by low-energy (LE), medium-energy (ME) and high-energy (HE) shown by green curves. The sum of the three fitted curves, shown in red, fits well with the measured PL profile. The whole PL spectra with various Bi compositions shown in Fig. 3(a) have been simulated with Gaussian fitting and the fitted energy peaks are summarized in Fig. 3(b) and other information listed in Table 2. The peaks shown by purple and green dots (1.24–1.41 eV) only exist in LT InP and very dilute InP_1−x_Bi_x_ samples (*x* ≤ 0.42%), and have been just discussed. Both HE and ME peaks exist almost in all InPBi PL spectra and are dominant, while the LE peak is resolved only for samples with *x* ≥ 1.95%. These three peaks will be discussed in detail below to unveil their physical origins.

## Discussion

The HE, ME and LE energy levels are within the range of 0.7–1.10 eV, much smaller compared with the InP band gap of 1.42 eV. The FWHM is typically around 130–280 meV, much broader than that of the InP band gap peak (~20 meV) as seen in Table 2. Therefore they are expected to come from spatially well confined deep levels^38^,^40^. The HE emission energy is found to monotonically decrease with Bi concentration from 1.05 eV down to 0.98 eV and shifts at ~31 meV/%Bi. The ME emission energy is found to monotonically decrease with Bi concentration from 0.95 eV down to 0.80 eV and shifts at ~80 meV/%Bi. Recent theoretical calculations by Kopaczek *et al*. reveal that the CB shift is 27 meV/%Bi and the VB shift is 79 meV/%Bi in InPBi^44^. Considering the HE and ME shift versus Bi concentration is close to the CB and the VB shift, respectively, we attribute the HE transition to electrons in the CB of InPBi recombining with holes trapped at a deep level and the ME transition to electrons trapped at a deep level to holes in the VB of InPBi.

As is shown in Fig. 3(a), when Bi doping into InP, the PL intensity is enhanced remarkably. Considering that the Bi atom is much larger than the P atom, it would be expected that the incorporation of Bi introduces defects, and thus degrade the material quality. In addition, the InP was grown at a significantly lower temperature than the optimal one, which will induce many defects such as P-related defects (P-interstitials and P-antisites) and In vacancies. These defects are expected to lead to non-radiative recombination. However, as is mentioned above, the introduction of the Bi impurities forms localized isoelectronic trap states near the valence band and they serve as trapping centers for the formation of bound holes. This spatial localization states smear out the electronic eigen-states in *k*-space and become delocalized in Brillouin zone. This proves advantages in allowing radiative transition from these states to the band edges and effectively reduces the loss to non-radiative centers. It is noteworthy that a similar mechanism has been proposed for the strong light emission from GaInN alloys^48^. It should also be taken into account that incorporating Bi during low temperature growth enhances adatom surface migration that will reduce the density of In- and/or P-related defects. However, as the Bi concentration increases, the Bi-related defects start to generate non-radiative centers and diminish the benefit. Thus, eventually high Bi concentrations lead to degradation of the optical quality of InPBi.

It has been shown that the band gap reduction, δ*E_g_*, in a semiconductor follows a scaling rule^49^:


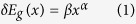


where *x* is the mole fraction of the constituent, *α* and *β* are two constants. The α -value is close to 1, 1/3 and 2/3 for regular semiconductor alloy, doped semiconductor and isoelectronic semiconductor, respectively. For a regular and completely miscible isoelectronic alloy with a small difference in electronegativity, virtual crystal approximation is valid and the band gap is simply a linear interpolation between the two constituent binaries. The band gap reduction is thus linear with the alloy composition and α = 1. For heavily doped *n*- or *p*-type semiconductor, the band gap reduction as a result of Mott transition is proportional to the carrier-carrier interaction characterized by the average impurity separation, resulting in the 1/3 power dependence. For isoelectronic alloy with a large difference in electronegativity and a large miscibility gap like N (Bi) in GaP or GaAs, a set of discrete impurity energy levels due to single N (Bi) atoms or N (Bi) pairs exist close to the conduction (valence) band. The strong interaction between the N (Bi) impurity level and the conduction (valence) band causes band gap anti-crossing and consequently the band gap reduction. The difference between N and Bi is that the former interacts with the conduction band and thus has a strong effect on electrons, while the latter affects the valence band and holes. The resulting alloy will trap electrons (for N) or holes (for Bi) with a potential much steep than *r*^−1^, where *r* is the distance from the trap, and become easily charged. For example, the neutral Bi_P_ trap has a binding energy of 40 meV for trapping a hole^48^, much larger than a few meV for most shallow donors or acceptors. This leads to the 2/3 power dependence.

**Figure 4:**
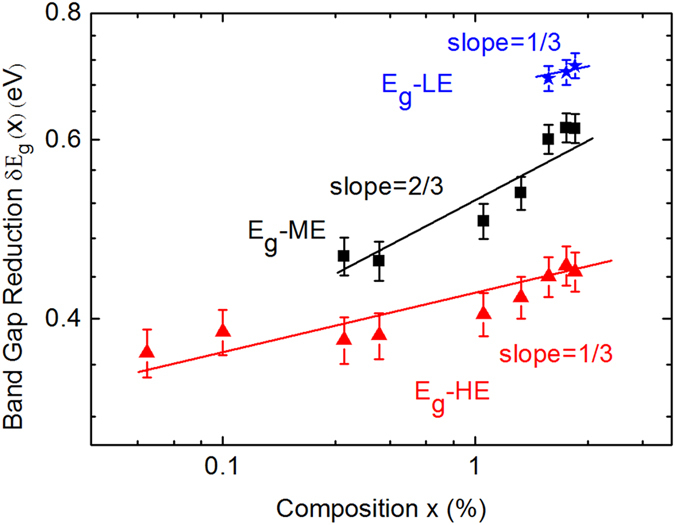
Band gap reduction as a function of Bi composition shown in log-log scale.

For the HE and ME found in InPBi, we define:


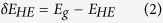



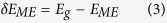


where *E*_*g*_ is the band gap of InP, *E*_*HE*_ and *E*_*ME*_ are the transition energy for HE and ME, respectively. Figure 4 shows the log-log plot of the band gap reduction in InPBi as a function of Bi composition. The two dominant deep level emissions show different scaling exponents: α_ΗΕ_  ≈ 1/3 and α_ΜΕ_  ≈ 2/3. The significance of the HE scaling exponent being closed to 1/3 confirms that the final state is primarily due to the formation of an impurity band associated with Bi-related defects. Meanwhile the significance of the ME scaling exponent being closed to 2/3 similar to GaAs:N implies that the ME emission is related to the VB anti-crossing raised by isoelectronic Bi doping as in the case of the CB lowered by isoelectronic N doping.

**Figure 5:**
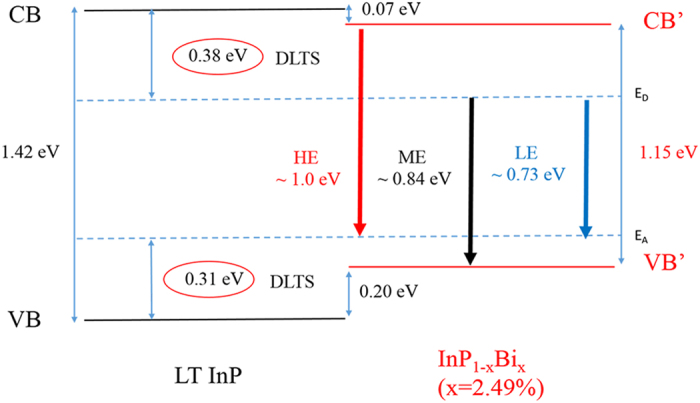
Energy diagram and the origin of InPBi HE, ME and LE PL emissions at low temperature. Two dashed lines are deep levels determined by DLTS.

In order to determine the nature of InPBi PL and verify the existence of deep levels, we performed deep level transient spectroscopy (DLTS) measurements on the LT InP reference and In_1−x_PBi_x_ (*x* = 2.49%) grown on *n* + InP substrates. From the DLTS results, two deep levels with the ionization energy of the *n*-type deep level to be 0.38 eV below the CB edge and the *p*-type deep level to be 0.31 eV above the VB maximum of InP, respectively, are identified. Base on the PL spectra and the DLTS results, the physical origins of HE, ME and LE can be explained as shown in Fig. 5 with InP_1−x_Bi_x_ (*x* = 2.49%) as example. At a low temperature, the InP band gap is equal to1.42 eV. According to the theoretical calculation^44^ mentioned above, the InPBi band gap should be 1.15 eV and shift by 0.07 eV and 0.20 eV from the CB and VB edge, respectively. Keeping these in mind and combing the results of DLTS, the CB to the *p*-type deep level HE emission energy is about 1.0 eV, the *n*-type deep level to the VB ME emission energy is about 0.84 eV, and the LE emission energy which only appears at high Bi concentrations is about 0.73 eV. These three deduced transition energy values agree well with the experimentally fitted PL peaks of 0.98 eV, 0.81 eV and 0.71 eV, respectively, at 10.5 K. From Fig. 4, it is easily proved that δ*E*_*LE*_ will follow


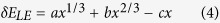


where *a*, *b* and *c* are three constants. For very small *x*-values, δ*E*_*LE*_ ≈ *ax*^*1/3*^, indicating that it also follows the 1/3 power dependence as δ*E*_*HE*_ which is true as shown by the blue line in Fig. 4. The 0.38 eV donor-like deep level is likely related to the intrinsic deep level in LT grown InP. Liang *et al*. found a deep level at 0.32 ± 0.05 eV below the CB in InP grown at 130–410 °C by MBE using admittance spectroscopy measurement^50^, which is close to the value from our DLTS measurement. This level is considered to originate from the intrinsic antisite of P_In_. The overdose of P flux compared with In at low growth temperature causes excess P atoms occupying In lattice sites forming P_In_ antisites and consequently resulting in high background *n*-type doping. While the VB of InPBi (red line in Fig. 5) results from the anti-crossing of the Bi impurity level with the VB of InP, we attribute the *p*-type deep level to the formation of Bi pairs or complex Bi related clusters. It has been theoretically shown that Bi pairs in GaP:Bi can form a series of energy levels above the VB^51^. The energy difference from the VB edge increases with decrease of the Bi pair distance and can reach as deep as 0.5 eV^51^. This is also expected in InP:Bi since the Bi impurity level is slightly below the VB edge of InP^52^. If Bi clusters with three or more Bi atoms and/or complex Bi clusters consisting of Bi atoms combined with antisite defects or P vacancies are formed, the trap energy is expected to be even large. Although it is difficult to map a deep energy level to a particular atomic configuration experimentally, our scanning tunneling microscopy (STM) measurements (not shown here) do reveal presence of such defects. The strong spatial localization of both *n*- and *p*-type deep levels found in InPBi translates into broad *k*-values in the momentum space, which explains the large FWHM of these transitions involving deep levels.

It is interesting to note the order of the appearance of HE, ME and LE transitions. The HE transition is already discerned for InPBi with *x* = 0.05% and its intensity increases with Bi concentration up to *x* = 0.42% and then decreases afterwards. The ME transition has a similar scenario for 0.30% ≤ *x* ≤ 2.49% followed by the LE transition appeared for *x* ≥ 1.95%. From Fig. 4, this means strong competition occurs between carrier populations at different energy levels. Time resolved PL is needed to further understand carrier dynamics and the Bi composition dependent PL behavior.

In summary, we have carefully studied low temperature PL of InP_1−x_Bi_x_ with various Bi concentrations (0% ≤ *x* ≤ 2.49%) and explained the origin of InP_1−x_Bi_x_ emission. The PL intensity is found to significantly increase by Bi doping due to the effective spatial trapping of holes, which is a positive indication for light emitting devices. The PL spectra evolution versus Bi concentration shows that features at high energy gradually quench and features at low energy emerge. Three dominate emission are resolved in PL and are related to two deep levels confirmed by DLTS: the E_D_ level of 0.38 eV below the bottom of CB due to intrinsic P_In_ defects and the E_A_ level of 0.31 eV above the top of VB associated with Bi-related defects, such as Bi clusters. Our findings open up the possibility to engineer the broad and strong luminescence for making super-luminescence diodes, which can theoretically enhance the spatial resolution beyond 1 μm in optical coherent tomography (OCT).

## Methods

### Molecular Beam Epitaxy

InP_1−x_Bi_x_ thin films of ~390 nm thick and with 0%≤ x ≤ 2.49% were grown directly on semi-insulating (100) InP substrates without InP buffer by the V90 GSMBE system. P_2_ was cracked from PH_3_ at 1000 °C. Its flux was controlled by regulating the gas pressure in the gas line. Elementary In and Bi sources were used and their fluxes were controlled by adjusting the effusion cell temperatures. Both substrate and cell temperatures were measured by thermocouples. For Bi incorporation, the growth temperature was decreased to about 320 °C after oxide desorption at 510 °C. The set of samples with different Bi contents were grown with a varying Bi:In BEP ratio (0–0.16) but a constant growth rate of 13 nm/min and a fixed PH_3_ pressure of 350 Torr. An InP reference sample was grown under the same growth condition (LT InP) for comparison.

**Figure 6:**
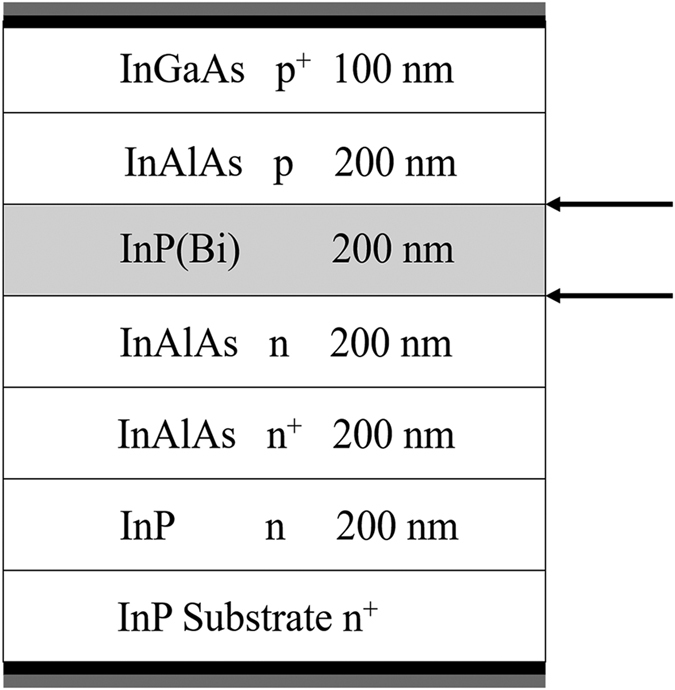
Diagram showing the layer structure of the *p-i-n* diodes for DLTS measurements of InP and InPBi. The arrows indicate a growth interrupt for growth temperature changes. The black and gray shaded areas at the top and bottom of the diagram indicate the Ohmic contacts.

InP_1−x_Bi_x_ (*x* = 0%, *x* = 2.49%) p-i-n structures were grown by MBE on *n*-type (100) InP substrate, as shown in Fig. 6. The n + /n/p-doped InAlAs and p^+^-doped InGaAs layers were grown at a growth rate of 16 nm/min and temperature of 412 °C. The *i*-layer were grown at a much lower temperature of 265 °C and a growth rate of 13 nm/min. The diodes were fabricated with large area Ti/Au Ohmic contacts on the backside of the substrate and 100 μm-diameter Ti/Au Ohmic contact to the p^+^ InGaAs cap layer.

### Measurements

Structural qualities were characterized by a Philips X’pert MRD high-resolution X-ray diffractometer (HRXRD). We have calculated both Bi concentration and strain relaxation using Vegard’s law from deduced perpendicular and in-plane lattice constants of the InPBi epilayer, which were determined by (004) symmetric ω-2θ rocking curves and (115) asymmetric ω-2θ rocking curves using the extrapolated InBi lattice constant of 6.52 Å^32^,^38^. The estimated Bi concentration from HRXRD is corroborated by Rutherford back scattering (RBS) measurements^38^.

The PL and absorption spectra were measured using a Fourier transform infrared (FTIR) spectrometer-based PL system^53^ in the rapid- rather than the step-scan mode^54^, in which a liquid-nitrogen cooled InSb detector and a CaF_2_ beam splitter were used. Laser of wavelength at 532 nm was used as the excitation. The samples were mounted into a close-cycle refrigerator for the low temperature PL measurements.

DLTS measurements were carried out by using the Semilab DLS-38D type deep-level transient spectroscopy. The temperature range for measurements was 20–320 K.

## Additional Information

**How to cite this article**: Wu, X. *et al*. Anomalous photoluminescence in InP_1−x_Bi_x_. *Sci. Rep.* **6**, 27867; doi: 10.1038/srep27867 (2016).

## Figures and Tables

**Table 1:** FWHM of the epitaxial layer peak for InP_1−x_Bi_x_ (0.30% ≤ *x* ≤ 2.49%).

Composition (%)	FWHM (arcsec) (±2 arcsec)
0.30	189
0.42	94
1.08	64
1.52	49
1.95	46
2.49	46

**Table 2:** Energy position and FWHM of the fitted HE, ME and LE for InP_1−x_Bi_x_ (0.05% ≤ *x* ≤ 2.49%).

Composition (%)	Energy position (eV) (±0.02 eV)			FWHM (eV) (±0.03 eV)		
	HE	ME	LE	HE	ME	LE
0.05	1.05	–	–	0.19	–	–
0.10	1.03	–	–	0.20	–	–
0.30	1.04	0.96	–	0.14	0.14	–
0.42	1.04	0.96	–	0.13	0.16	–
1.08	1.02	0.92	–	0.13	0.19	–
1.52	1.0	0.89	–	0.14	0.24	–
1.95	0.98	0.82	0.73	0.14	0.20	0.28
2.30	0.97	0.80	0.72	0.14	0.14	0.20
2.49	0.98	0.81	0.70	0.13	0.13	0.16
